# Preclinical safety evaluation of low molecular weight galactomannans based standardized fenugreek seeds extract

**DOI:** 10.17179/excli2016-461

**Published:** 2016-07-13

**Authors:** Pallavi Deshpande, Vishwaraman Mohan, Prasad Thakurdesai

**Affiliations:** 1 Indus Biotech Private Limited, 1, Rahul Residency, Off Salunke Vihar Road, Kondhwa, Pune 411048, India

**Keywords:** low molecular weight galactomannans, Fenugreek seed extract, acute oral toxicity, subchronic toxicity, mutagenicity, OECD guidelines

## Abstract

The objective of the present study was to evaluate acute oral toxicity, subchronic toxicity, and mutagenic potential of low molecular weight galactomannans based standardized fenugreek seeds extract (LMWGAL-TF) in laboratory animals rats as per Organization for Economic Co-operation and Development (OECD) guidelines. For the acute toxicity (AOT) study, LMWGAL-TF was orally administered to Sprague-Dawley (SD) rats at a dose of 2000 mg/kg with vehicle control (VC) group (n = 5 per sex per group) as per OECD guideline no. 423. For the repeated dose toxicity study, the SD rats were orally administered with a daily oral dose of LMWGAL-TF 250, 500 and 1000 mg/kg/day with VC group (n = 15 per sex) for a period of 90 days followed by a recovery period of 28 days as per OECD guideline no. 408. The effects on body weight, food and water consumption, organ weights with hematology, clinical biochemistry, and histology were studied. The mutagenic potential of LMWGAL-TF was tested using reverse mutation assay (AMES test, OECD guideline No. 471). The LMWGAL-TF did not show mortality or treatment-related adverse signs during acute (dose 2000 mg/kg) and subchronic (90-days repeated dose 250, 500 and 1000 mg/kg) administration. The LMWGAL-TF showed oral lethal dose (LD_50_) more than 2000 mg/kg during AOT study. The dose of 1000 mg/kg was found as no observed adverse effect level (NOAEL) in rats during subchronic toxicity study. Furthermore, LMWGAL-TF did not show mutagenic potential in vitro. In conclusion, LMWGAL-TF was found safe during acute and subchronic (90 days repeated dose) toxicity studies in rats with no mutagenicity.

## Introduction

Since ancient times, plants (or food) derived natural products have commonly been used in folk medicine for the treatment of various ailments. The growing use of plant-derived food supplements is accompanied by an increasing concern for their safety (Werner, 2014[23]). Therefore, scientific data related to the safety evaluation of plant-derived food supplements has become crucial for validation of quality and safety claims for their safe human consumption (Govindaraghavan and Sucher, 2015[6]). 

Because of a broad spectrum of therapeutic benefits, the fenugreek (*Trigonella foenum-graecum* L., family Fabaceae) seeds have received considerable attention as plant-derived food / nutritional supplement (Yadav and Baquer, 2014[27]). Fenugreek seeds are documented in many traditional systems of medicine, such as Ayurveda, for the management of many chronic conditions. The modern scientific literature reported beneficial effects of fenugreek seeds powder and extract on glucose and fat metabolism in animal models and patients with diabetes mellitus (Roberts, 2011[20]). Fenugreek seed derived food supplement products including extracts have been explored successfully for many exercise physiology applications involving healthy volunteers and patients (Basch et al., 2003[2]; Thompson and Ernst, 2003[21]) including anabolic (Poole et al., 2010[19]) and androgenic activities (Mokashi et al., 2014[11]; Wilborn et al., 2010[25]). Safety of fenugreek seeds in human subjects has been reported in many clinical studies and reviews (Basch et al., 2003[2]; Poole et al., 2010[19]; Thompson and Ernst, 2003[21]). 

Recently, low molecular weight galactomannans-based standardized fenugreek seed extract (LMWGAL-TF) showed promising and dose-dependent efficacy in an animal model of hyperglycemia and insulin resistance (Kandhare et al., 2015[9]). In addition, LMWGAL-TF had demonstrated anabolic ability without affecting hormonal status in animals (Aswar et al., 2008[1]) and the exercising individuals (Wilborn et al., 2008[24]). However, safety information of LMWGAL-TF using scientifically validated and internationally accepted guidelines is lacking. Therefore, we undertook the present work with an objective to evaluate the acute oral toxicity (AOT), 90-days repeated dose (subchronic) toxicity and mutagenicity of LMWGAL-TF using well-accepted guidelines issued by Organization for Economic, Co-operation, and Development (OECD).

## Materials and Methods

### Animals

The Sprague-Dawley rats; 6-8 weeks old weighing 125-150 g of both sexes were used for acute and 90-day (subchronic) toxicity studies. The females were nulliparous and non-pregnant. The rats kept for seven days prior to dosing for acclimatization, cages were marked, and individual marking was made on fur for identification. Rats were on pelleted feed (Nav Maharashtra Chakan Oil Mills Ltd, Pune, India) and provided with pure potable water in glass bottles *ad labium*. The rats were maintained at ambient temperature (20 ± 3° C), relative humidity (30-70 %), and 10-15 air changes per hour with 12 h: 12h of the dark and light cycle. 

All experiments complied with the OECD Guidelines for the testing of chemicals (Organisation for Economic Co-operation and Development, 1998[13]) and Schedule Y in Drugs and Cosmetics Act (II^nd^ Amendment) Rules, 2005, Ministry of Health and Family Welfare, Government of India. The protocol was approved by the Institutional Animal Ethics Committee.

### The test compound

The test compound, LMWGAL-TF, was supplied by Indus Biotech Private Limited (Pune, India) after preparation and characterization (HPLC and LC-MS) as per reported procedure (Kamble et al., 2013[8]). LMWGAL-TF is standardized fenugreek seed extract with 96.21 % water-soluble low molecular weight galactomannan containing oligosaccharides. LMWGAL-TF is commercially available as Torabolic^®^. LMWGAL-TF was dissolved in distilled water for use in acute and sub-chronic studies in a dose volume of 10 ml/kg body weight of rats. LMWGAL-TF solution was freshly prepared before administration. LMWGAL-TF solution in sterile water for injection was used for mutagenicity evaluation. 

### Acute oral toxicity (AOT) study 

The acute toxicity study of LMWGAL-TF was performed according to OECD guideline 423 (Organisation for Economic Co-operation and Development, 2002[16]). The SD rats were grouped in 2 groups (5 rats/sex/group) and treated with a single dose as follows: G1 - Vehicle control (VC) (double distilled water, 10 mL/kg, oral), and G2 (LMWGAL-TF, 2000 mg/kg, oral.). The rats were observed daily for 14 days following administration for mortality and clinical signs of toxicity. On day 15, all animals were euthanized and underwent gross pathological examination for signs of toxicity via necropsy.

### Repeated dose 90-day oral toxicity (Sub-chronic) study

The study complies with the OECD Guideline for the testing of Chemicals No. 408 (Organisation for Economic Co-operation and Development, 1998[15]). Based on median lethal dose (LD_50_) (2000 mg/kg) obtained from AOT study, animals were orally administered daily once with following treatments:

- G1 - VC (Distilled water, 10 mL/kg, 90 days) - 15 animals per sex
- G2 - LMWGAL-TF-250 (LMWGAL-TF, 250 mg/kg, 90 days) - 15 animals per sex
- G3 -LMWGAL-TF-500 (LMWGAL-TF, 500 mg/kg, 90 days) - 15 animals per sex
- G4 - LMWGAL-TF-1000 (LMWGAL-TF, 1000 mg/kg, 90 days) - 15 animals per sex
- G1R - VC reversal group -VC-R - (Distilled water, 10 mL/kg, 119 days) - 10 animals per sex
- G4R - LMWGAL-TF-1000-Reversal group (LMWGAL-TF-1000-R (LMWGAL-TF, 1000 mg/kg for 90 days) followed by (distilled water, 10 mL/kg (from day 91 to day 119) - 10 animals per sex

All treated rats were observed daily for mortality and clinical signs during the 90-day study period whereas reversal groups (G1R and G4R) were observed for up to day 119 (Reversal period of 28 days). The eyes of control and all the treated dose group animals were examined prior to the initiation of the dosing and on day 91 and 119 (for G1R and G4R) of the study. Eye examination was carried out using an ophthalmoscope (mini 2000, HEINE Optotechnik, Herrsching, Germany) after induction of mydriasis with 0.5 % solution of tropicamide. The body weight and feed consumption of each rat were recorded at weekly intervals throughout the study period. Towards the end of the exposure period (day 91), functional observations (by grading different sensory reactivity to stimuli of different types (auditory, visual and proprioceptive stimuli), assessment of grip strength (digital grip strength meter (Columbus Instruments International, Columbus, OH, USA)) and motor activity measurement were performed. The animals were placed in metabolic cages overnight and urine excreted by each animal was collected on day 91 and on day 119 (G1R and G4R) of the study for urinalysis. The parameters for urinalysis included: specific gravity, pH, occult blood, protein, bilirubin, ketones, glucose, nitrite, and urobilinogen. Using whole blood, hematological and coagulation analyzes were carried out. The parameters for hematological analysis included: red blood cell count (RBC), reticulocyte count, hemoglobin (Hb), hematocrit (HCT), mean corpuscular hemoglobin (MCH), mean corpuscular hemoglobin concentration (MCHC), mean corpuscular volume (MCV) and total leukocyte count (TLC), % cells in differential leukocyte count (DLC) including lymphocyte (L), monocyte (M), basophils (B), eosinophils (E), and Prothrombin time (PT). Additionally, clinical chemistry was evaluated including Blood Urea Nitrogen (BUN), Alanine Aminotransferase (ALT), Aspartate Aminotransferase (AST), Alkaline Phosphatase (ALP), Gamma Glutamyl Transferase (GGT), Creatine Phosphokinase (CK), Lactate Dehydrogenase (LDH), Fasting plasma glucose (FPG), Calcium (CA), Phosphorus (P), Bilirubin, Albumin, creatinine (CR), Sodium (Na), Potassium (K), Chlorine (Cl), Total cholesterol (TC), and Triglycerides (Trig). On day 91, all animals were euthanized and underwent gross pathological examination for signs of toxicity via necropsy. All organs, mucosa, body cavities, etc. were examined for gross pathological changes. Major organs and major endocrine glands (pituitary, adrenal, thymus, thyroid, sex, etc.) were weighed as absolute values and from that their relative values (i.e. percent of the body weight) were calculated. Tissue samples from selected organs (heart, kidney, liver, lung, spleen, stomach, pancreas and skeletal muscle) from the VC (G1 and G1R) and 1000 mg/kg (G4 and G4R) groups were preserved, fixed, and stained for histopathological evaluation via light microscopy. The remaining tissues/organs were preserved in 10 % neutral buffered formalin from control and different dose groups.

### Mutagenicity study

Mutagenicity was evaluated by bacterial Reverse Mutation test (AMES test) was performed in full compliance with the OECD guidelines for mutagenicity testing namely Test No. 471 (Organisation for Economic Co-operation and Development, 1997[17]). As no significant cytotoxic effect was observed, the five highest doses were then used in the subsequent mutagenicity evaluation. To evaluate mutagenicity, five strains of histidine-dependent *Salmonella typhimurium* (TA97a, TA98, TA100, TA1535 and TA102) were tested in triplicate at the five highest doses (5000.00, 1666.67, 555.55, 185.18 and 61.72 µg/plate) of LMWGAL-TF. Rat liver homogenate tested with mutagen 2-aminofluorene before use. Metabolic activation was performed using a cofactor supplemented post-mitochondrial fraction (S9 fraction). Positive controls (2-aminofluorene, 2-aminoanthracene, methyl methanesulfonate, 4-Nitroquinolene-N-Oxide, danthron, and sodium azide) with and without S9 activator and negative controls (VC and phosphate buffer) with and without S9 activator were included in the evaluation. This was done to ensure the test system was functioning properly (positive controls) and to obtain baseline revertant frequencies for the various strains of bacteria used in the study (negative controls). The plates were counted after 48 h of incubation at 37° C. The mutagenic activity of the test substance was considered for positive in case of increased concentration over the range tested and a reproducible increase at one or more concentrations in a number of revertant colonies per plate in at least one strain with or without metabolic activation system. The test substance was considered to be toxic if there was a decrease in the number of revertants and/or thinning or absence of background lawn.

### Statistical analysis

Statistical analysis was performed using SPSS analysis program for windows Version 17.0 (SPSS Inc, USA). All the data were checked for normality and values were represented as mean ± standard deviation (SD). Data of body weight and food intake was analyzed by two-way ANOVA followed by unpaired 't' test. Remaining parameters were analyzed by separate One way ANOVAs. Dunnett's test was used to analyze the differences between treated groups with respective VC groups (G2, G3, G4 v/s. G1 and G4R v/s.G1R). The value of P < 0.05 was considered to be statistically significant.

## Results

### Acute oral toxicity (AOT) study

There were not either deaths or treatment-related clinical signs of toxicity observed during the evaluation period nor was any weight loss observed in any animals. Finally, no treatment-related gross pathological changes were observed during necropsy. The results of this evaluation show that the single oral dose resulted in an LD_50_ of greater than 2000 mg/kg of body weight. 

### Repeated dose 90-day oral toxicity (Sub-chronic) study

No mortality was observed in any of the groups during the 90-day evaluation period. There were no treatment-related clinical signs of toxicity or ophthalmoscopic or functional abnormalities during the evaluation period. There were no abnormalities in functional observation battery parameters during handling (piloerection, reaction to removal, reaction to handling, palpebral closure, eye examinations, lacrimation, salivation, mucous membrane) and open field test (appearance, gait, mobility, arousal, respiration, tonic or clonic or stereotype movement, vocalization, rearing, urination, defecation).

**Figure 1:**
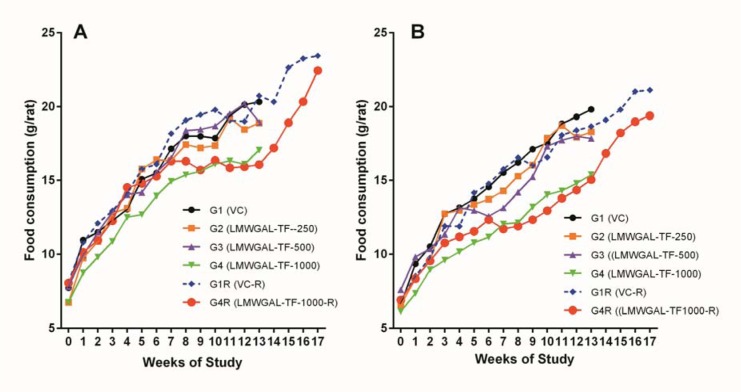
Effect of LMWGAL-TF on food consumption in (A) male and (B) female rats during 90 days repeated dose toxicity study. Data are expressed as mean ± Standard deviation (SD)

There were no statistically significant differences in average daily food consumptions in treatment groups as compared with respective VC groups during treatment or reversal period in male and female rats (Figure 1(Fig. 1)). Body weights of male and female rats did not show significant difference during the treatment period (Table 1(Tab. 1)). However, LMWGAL-TF-1000-R groups showed significant differences in body weights as compared with corresponding VC groups (P < 0.01 and P < 0.05 for male and female rats respectively) during reversal period. 

Hematological (Table 2(Tab. 2) and Table 3(Tab. 3)) and biochemical observations (Table 4(Tab. 4) and Table 5(Tab. 5)) and urinalysis were recorded in male and female rats. All the values hematological and biochemical values were within normal biological and laboratory limits and the differences between the values were not consistent with treatment doses or period of observation (treatment / reversal) and so not treatment specific. For example, in male rats, LMWGAL-TF-250 and LMWGAL-TF-500 group (but not LMWGAL-TF-1000) showed a significant decrease (P < 0.01) in % MCHC during the treatment period as compared with VC group. On the other hand, LMWGAL-TF-1000-R group showed significant increase in % MCHC (P < 0.01) and % MCH (P < 0.05) as compared with VC-R group. The HCT and RBC count did not change significantly between the treatment groups and VC in both sexes whereas significant decreases (P < 0.05) was found were found in LMWGAL-TF-1000 group as compared with VC-R group in female rats. On the other hand, platelet count of LMWGAL-TF-1000 group showed a significant increase (P < 0.05) as compared with VC group during treatment period but showed no significant change in LMWGAL-TF-1000-R group as compared to VC-R group in the reversal period in female rats. In case of blood biochemical parameters, AST values showed a significant (P < 0.05) decrease in LMWGAL-TF-1000-R as compared to VC-R group in female rats but male rats showed no statistical difference in LMWGAL-TF treated groups with their respective VC groups. Similarly, values of electrolytes Ca, Na, K and Cl showed significant changes between LMWGAL-TF treated groups during treatment and reversal group without consistent correlation with dose levels during treatment or reversal period.

**Figure 2:**
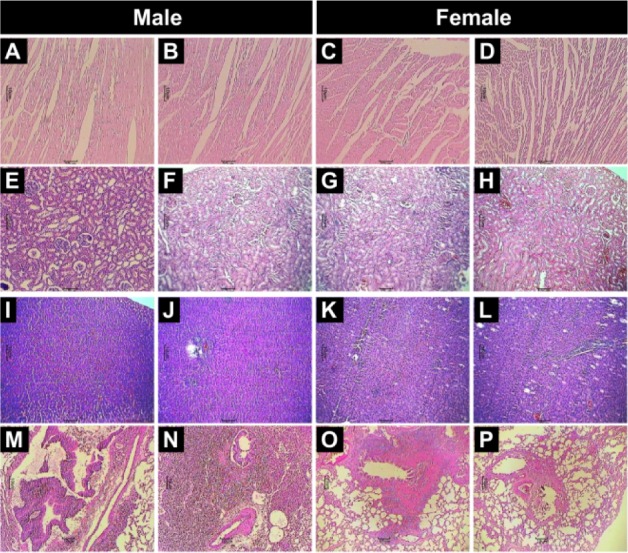
Effects of LMWGAL-TF on histological findings of heart (A-D), kidney (E-H), liver (I-L) and lung (M-P) tissue in rats during 90- days repeated dose toxicity study. Photomicrographs from representative rats from respective groups: Male: VC (A, E, I and M), LMWGAL-TF-1000 (B, F, J and N), Female: VC (C, G, K and O) and LMWGAL-TF-1000 (D, H, L and P) (H&E stain) at 40X

**Figure 3:**
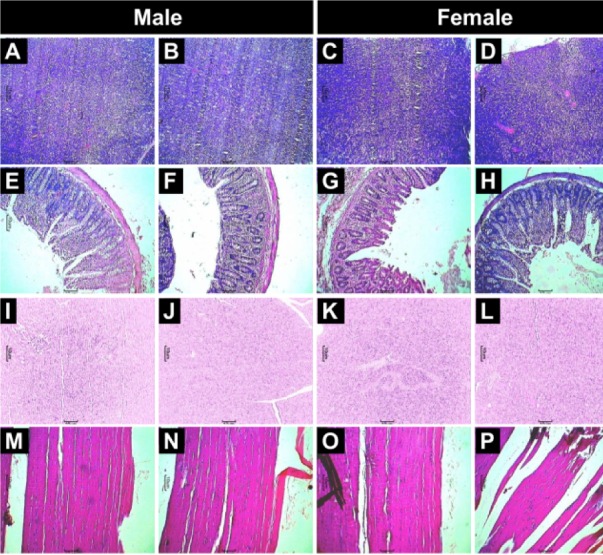
Effect of LMWGAL-TF on histological findings of spleen (A-D), stomach (E-H) and pancreas (I-L) and skeletal muscle (M-P) tissue in rats during 90 days repeated dose toxicity study. Photomicrographs from representative rats from respective groups: Male: VC (A, E, I and M), LMWGAL-TF-1000 (B, F, J and N), Female: VC (C, G, K and O) and LMWGAL-TF-1000 (D, H, L and P) (H&E stain) at 40X

No treatment-related gross pathological changes were observed in any organs of the test animals during necropsy. The data of weights of organs in LMWGAL-TF treated rats during treatment and reversal period in male and female rats is presented (Table 6(Tab. 6) and Table 7(Tab. 7)). None of the LMWGAL-TF treated group show significant difference during treatment or reversal period as compared with corresponding VC group. The finding from histological examination of sections of organs is presented in Table 8(Tab. 8) and representative photomicrographs are presented as Figure 2(Fig. 2) and Figure 3(Fig. 3). The changes observed in both VC and LMWGAL-TF-1000 treatment groups were similar and comparable in both sexes and hence considered as incidental, congenital, and spontaneous. Based on the results of present study, the no observed adverse effect level (NOAEL) is 1000 mg/kg per day (the highest dose level).

### Mutagenicity study

The bacterial background lawn was comparable with that of the respective VC plate up to the highest concentration of 5000 µg/ plate. No substantial increases in the revertant colony count in any of the five strains were reported at any of the test concentrations in the presence or absence of metabolic activation (S9 mix). Positive controls resulted in significant increases in the revertant count. The spontaneous reversion rates in the negative and positive control were within the range of historical data. The biologically relevant increase in the revertant counts was not observed in any of the five tester strains pre-incubated with the LMWGAL-TF. LMWGAL-TF did not induce gene mutation by pair changes or frameshifts in the genome of strains used during the present study.

## Discussion

In recent past, LMWGAL-TF has shown excellent potential for many therapeutic and nutritional applications for healthcare and sports nutrition area (Aswar et al., 2008[1]; Kandhare et al., 2015[9]; Visnagri et al., 2013[22]; Wilborn et al., 2008[24]). However, the safety and/or toxicology information on LMWGAL-TF has not been available. The present study evaluated LMWGAL-TF for acute and long term (90 days of daily administration) toxicity and mutagenicity potential in rats. Typically, safety studies on natural food supplements help to determine the dose levels for short-term and long-term repeated dose toxicity studies. The OECD guidelines are well-accepted standard for safety or toxicity assessments of medicinal. Therefore, the present study followed OECD guidelines. 

AOT data is considered valuable in establishing target organ toxicity. Repeated exposure of test substance over a long period of time has been utilized for the determination of adverse effects of a test substance qualitatively and quantitatively in laboratory animals (Joshua Allan et al., 2007[7]). In the present investigation, single oral administration of LMWGAL-TF showed median lethal dose (LD_50_) level of 2000 mg/kg. 

In the OECD guidelines for subacute oral toxicity assessment, the adverse effect of the test substances has been determined and NOAEL is determined by using various hematological, biochemical and histopathological analysis (Organisation for Economic Co-operation and Development, 1998[13]). Hence, we conducted sub-chronic 90-day repeated dose toxicity study. During the sub-chronic (90 days repeated dose) toxicity study, all rats survived until the scheduled euthanasia and no gross pathological alteration was found in the internal organs. A trend towards decrease (not statistically significant) in body weights in LMWGAL-TF groups that was observed during the present study is in line with the past reports of water-binding activity of oligosaccharides, slow gastric emptying, and increased bowel viscosity and subsequently decreased appetite and weight loss (Gentilcore et al., 2011[5]). In the past, many appetite suppressants were withdrawn from the market due to an increased risk of psychiatric disorders (Derosa and Maffioli, 2012[4]; Kang and Park, 2012[10]). However, LMWGAL-TF treated rats showed no significant changes in food consumptions during the present study. In addition, no abnormalities were observed in LMWGAL-TF group during treatment or reversal period during functional observation battery parameters during handling and open field test. Reversal groups (G1R and G4R) were incorporated to assess the reversibility of any potential toxicity that can progress after cessation of administration. G4R group did not show abnormalities or deviations from normal in physiological values after cessation of treatments in reversal groups Taken together, body weight loss (although not significant) in LMWGAL-TF treated groups indicate potential for development of LMWGAL-TF as a safe product without central nervous system side effects. 

Histopathological and biochemical findings did not find any abnormalities or deviations from normal physiological limits in LMWGAL-TF treated groups (G2, G3 and G4) compared to VC group at dose levels up to 1000 mg/kg. In the present study, LMWGAL-TF administration showed decreased MCH and MCHC without changes in Hb levels. However, these alterations were not sex- or dose-dependent and all values were within normal physiological range for rats (Wolford et al., 1986[26]). Hence, the changes were not related to LMWGAL-TF treatment and dose of 1000 mg/kg (highest tested dose) was considered as no observed adverse event level (NOAEL) for the subchronic administration of LMWGAL-TF in rats. 

A biological assay to evaluate the mutagenic potential of chemical compounds is AMES test (Mortelmans and Zeiger, 2000[12]). LMWGAL-TF was evaluated over a broad concentration range (61.72-5000 µg/plate) and did not exhibit signs of significant mutagenicity either in the presence or absence of a metabolic activator during the present study.

An array of studies established a good correlation between the efficacy and safety evaluation of various plant-based products and/or phytochemicals via toxicological properties in rats (Delaney, 2007[3]; Olson et al., 2000[18]). A previous study carried out by a researcher showed the efficacy of oral administration of LMWGAL-TF in an animal model of metabolic syndrome (Kandhare et al., 2015[9]) and diabetes mellitus (Kamble et al., 2013[8]) at 60 mg/kg/day (90-days) and 50 mg/kg/day (for 21 days) respectively. The difference between preclinical efficacy dose (60 mg/kg) and NOAEL (1000 mg/kg) of LMWGAL-TF indicate excellent margin of safety.

## Conclusion

The LD_50_ of LMWGAL-TF was found to be more than 2000 mg/kg. The NOAEL of LMWGAL-TF was found as 1000 mg/kg in both male and female rats. The present study indicated the safety for development of agent for clinical management of chronic disorders after appropriate clinical studies.

## Acknowledgements

We acknowledge Indian Institute of Toxicology, Pune, India for their contract research services. 

## Conflict of interest

The authors declare no conflict of interest.

## Figures and Tables

**Table 1:**
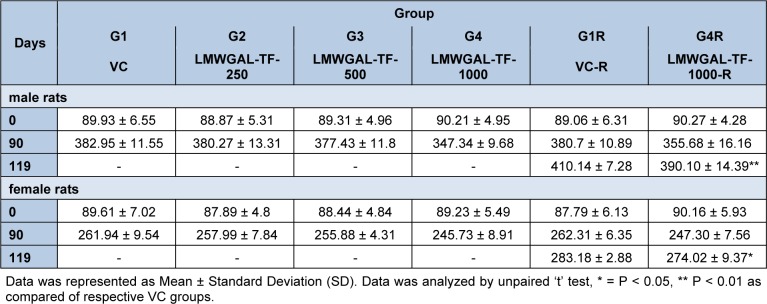
Effect of LMWGAL-TF on body weights (g) of rats during 90 days repeated dose toxicity study

**Table 2:**
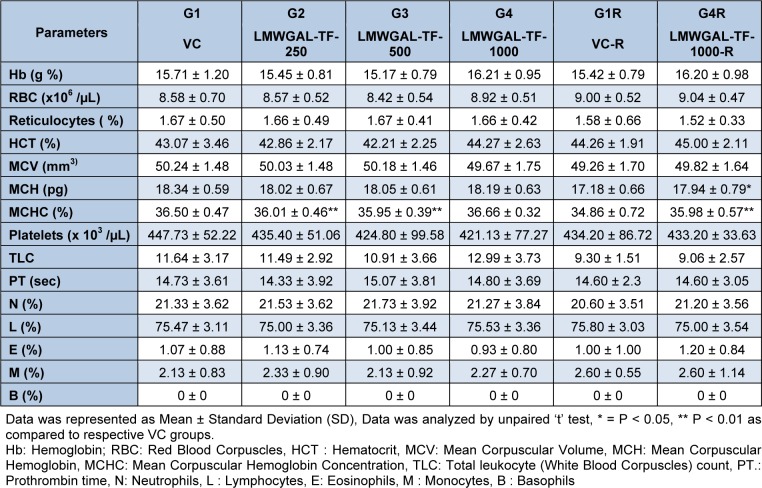
Effect of LMWGAL-TF on hematological parameters during 90 days repeated dose toxicity study (male rats)

**Table 3:**
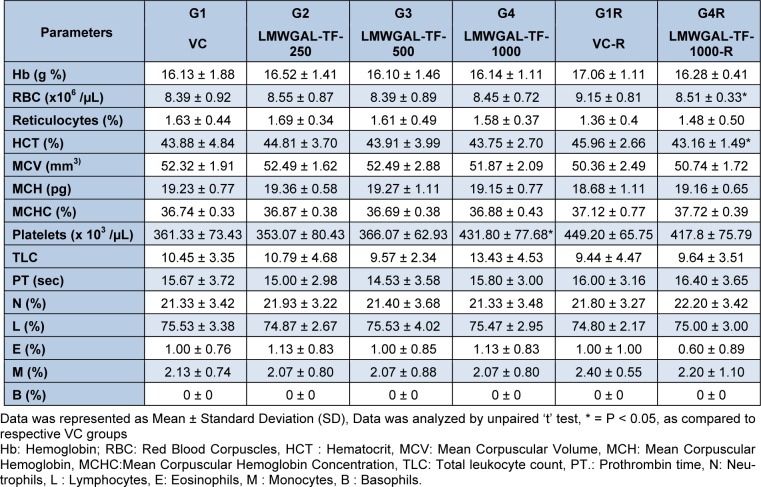
Effect of LMWGAL-TF on hematological parameters during 90 days repeated dose toxicity study (female rats)

**Table 4:**
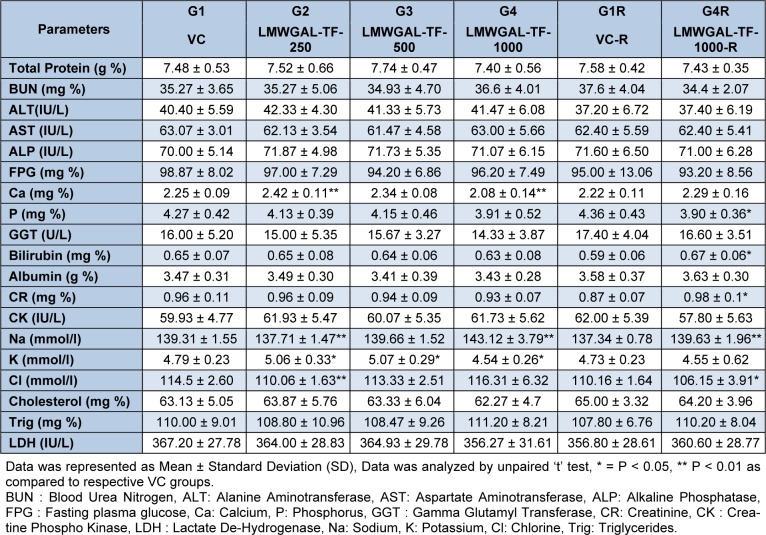
Effect of LMWGAL-TF on blood biochemistry on during 90 days repeated dose toxicity study (male rats)

**Table 5:**
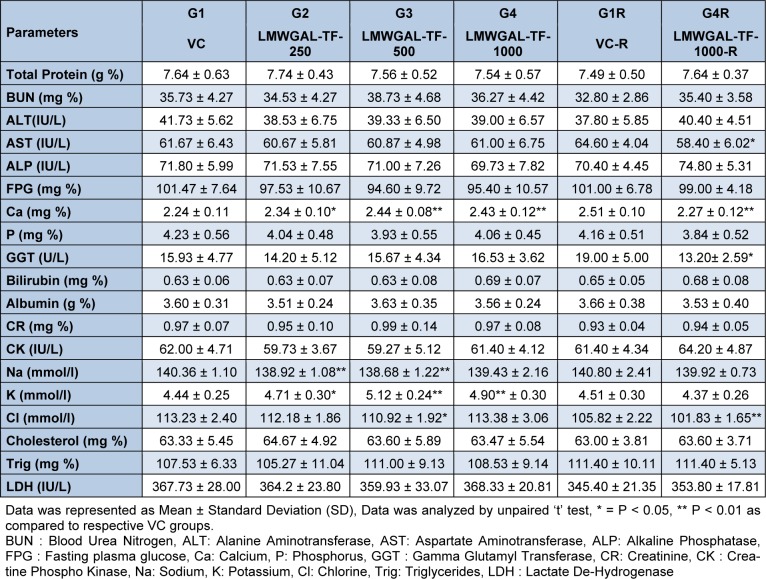
Effect of LMWGAL-TF on blood biochemistry on during 90 days repeated dose toxicity study (female rats)

**Table 6:**
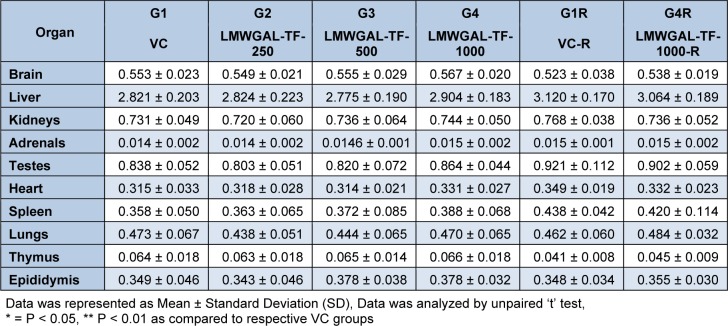
Effect of LMWGAL-TF on organ weights (g) of during 90 days repeated dose toxicity study (male rats)

**Table 7:**
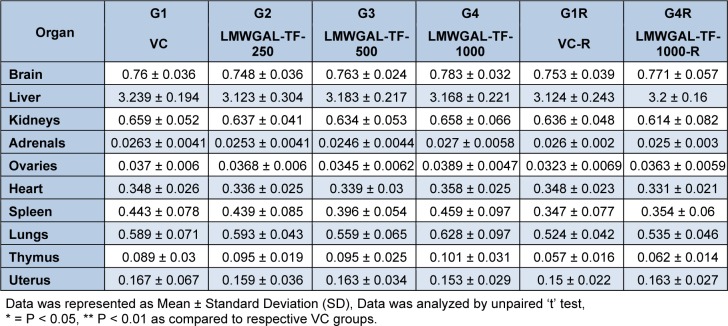
Effect of LMWGAL-TF on organ weights (g) of during 90 days repeated dose toxicity study (female rats)

**Table 8:**
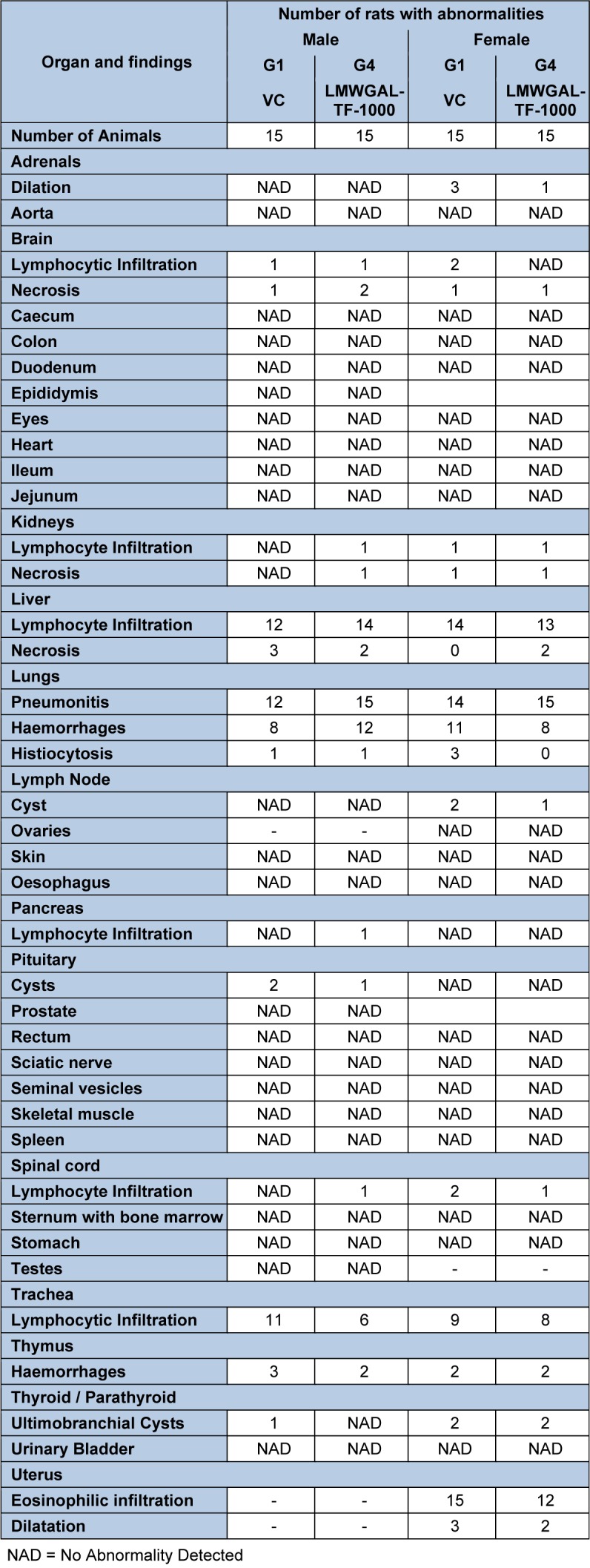
Summary of histopathology findings
